# The fungal STRIPAK complex: Cellular conductor orchestrating growth and pathogenicity

**DOI:** 10.1371/journal.ppat.1013500

**Published:** 2025-09-15

**Authors:** Patricia P. Peterson, Joseph Heitman

**Affiliations:** Department of Molecular Genetics and Microbiology, Duke University Medical Center, Durham, North Carolina, United States of America; University of Michigan, UNITED STATES OF AMERICA

## The STRIPAK complex is a conserved signaling module in eukaryotes

The striatin-interacting phosphatase and kinase (STRIPAK) complex is a highly conserved, multi-subunit signaling module present throughout the eukaryotic kingdom, from fungi to mammals. The core of STRIPAK is composed of protein phosphatase 2A (PP2A), a serine/threonine phosphatase responsible for major cellular phosphatase activity, which typically functions as a heterotrimeric enzyme composed of a scaffold A subunit, a regulatory B subunit, and a catalytic C subunit. Among the four known classes of B subunits, the B″′ family, also known as striatins, serves as the hallmark STRIPAK component. When striatin functions as the regulatory subunit, additional interacting partners are recruited to form the larger, multi-modular STRIPAK complex [1–3].

**Fig 1:**
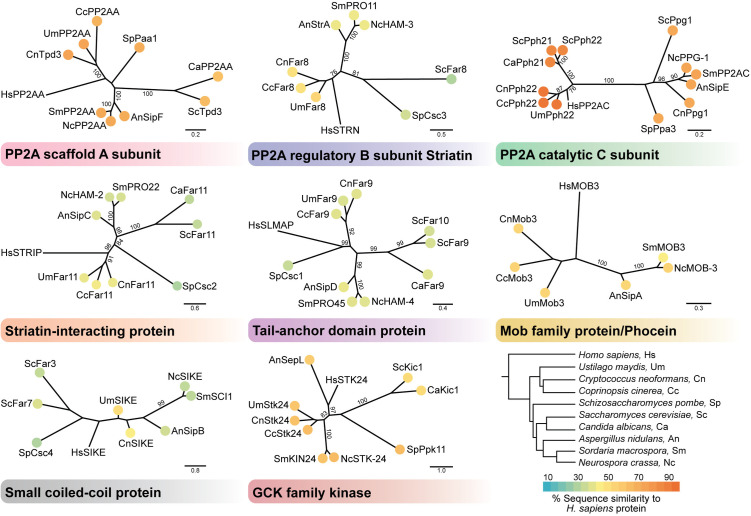
Phylogenetic analysis of striatin-interacting phosphatase and kinase complex subunits across fungi and humans reveals deep evolutionary conservation. The STRIPAK complex is composed of conserved subunits, including the scaffold A subunit (Tpd3/PP2AA), regulatory B subunit (Far8/STRN), and catalytic C subunit (Pph22/PP2AC) of protein phosphatase 2A (PP2A), along with several associated proteins: a striatin-interacting protein (Far11/STRIP), a tail-anchored membrane protein (Far9/SLMAP), a Phocein subfamily protein (Mob3), a small coiled-coil protein (SIKE), and a GCK family kinase (Stk24). To assess conservation across species, maximum likelihood phylogenies were constructed for each subunit with homologs identified in *Homo sapiens* and nine fungal species. These fungi include representatives from both the Ascomycota and Basidiomycota phyla, enabling comparisons across the major Dikarya clade of the fungal kingdom. A species-level phylogeny, based on established taxonomy, is shown for reference. Terminal nodes are colored according to percent sequence similarity to the human homolog. For the PP2A catalytic subunit, some species contain two paralogs (for example, Pph21 and Ppg1), both of which may participate in the STRIPAK complex. In some cases, multiple homologs have been identified for a given component, or the component was not found [21]. These patterns reflect both conservation and diversification of STRIPAK complex composition across eukaryotes. Trees were generated from trimmed MAFFT alignments using RAxML with the LG substitution model, 1,000 bootstrap replicates, and an extended majority-rule consensus approach. Bootstrap values >75 are indicated. Scale bars represent the number of amino acid substitutions per site.

STRIPAK was first discovered in mammalian cells by high-affinity purification approaches that mapped interactions between the PP2A A/C dimer with the striatin B subunit, Ste20-like kinases, and other regulatory components [2]. Subsequent studies revealed that STRIPAK controls a wide spectrum of signaling and developmental processes, including cell migration, polarity, cell cycle progression, apoptosis, neural and vascular development, and vesicular trafficking [3–7]. Homologous STRIPAK complexes have since been identified in diverse fungal organisms, including both saprophytic and pathogenic species, highlighting the deep evolutionary conservation of both its structure and function [8–11] (Fig 1). Despite organism-specific adaptations, the core components and their roles in phosphorylation-dependent signaling remain remarkably conserved, underscoring STRIPAK’s central role in coordinating cellular responses to environmental stimuli and developmental cues. This review summarizes our current knowledge of STRIPAK complexes in fungi, with a focus on their key roles in signaling networks that govern growth, differentiation, and pathogenesis.

## What roles does STRIPAK play in fungal development and morphogenesis?

Fungi serve as excellent models for studying developmental processes due to their distinct cell morphologies throughout sexual and asexual life cycles. Cellular fusion during mating, subsequent hyphal formation, and the production of mature sexual fruiting bodies are tightly coordinated processes during sexual development in most fungi. In filamentous fungi, following karyogamy (nuclear fusion) and meiosis, sexual spores are produced from highly complex multicellular structures. Studies in *Sordaria macrospora* and *Neurospora crassa* revealed that STRIPAK components are required for proper execution of these developmental events. Screens for mutants with defects in specific stages of the sexual cycle identified mutations in genes encoding STRIPAK complex subunits, highlighting the complex’s central role in sexual differentiation [11,12]. In several species of the plant pathogen *Fusarium*, mutations in the striatin homolog *FSR1* also disrupt sexual reproduction [13].

In addition to STRIPAK’s role in sexual development, it is also important for vegetative growth and asexual reproduction. In *Aspergillus nidulans,* deletion of STRIPAK components results in defects in hyphal cell fusion, conidiophore formation, and conidial spore production [14]. STRIPAK is also involved in regulation of cell polarity, a key determinant of hyphal extension and directional growth. STRIPAK complex mutants display disorganized or hyperbranched hyphae, suggesting a role in spatial control [15]. Similar phenotypes have been observed in *N. crassa* and *S. macrospora*, where STRIPAK mutants show impaired hyphal elongation and defects in septation and cell fusion during vegetative growth [11,16,17]. In the basidiomycete human pathogen *Cryptococcus neoformans*, deletion mutants lacking STRIPAK complex subunits also exhibit defects in mating, hyphal formation and elongation, and sporulation [18]. A recent study has further revealed that disruption of STRIPAK alters the mode of sexual reproduction in *C. neoformans* [19]. Specifically, mutants in STRIPAK components *PPH22*, which encodes the catalytic subunit of PP2A, and *FAR8*, which encodes the regulatory B subunit (striatin), undergo pseudosexual reproduction [20], with progeny inheriting nuclear genomes exclusively from the wild-type parent, implicating STRIPAK in nuclear inheritance and meiotic progression. Notably, these mutants also exhibit widespread aneuploidy, suggesting that STRIPAK is required for genome stability [18]. These findings highlight the role of STRIPAK in maintaining genome integrity and coordinating nuclear dynamics during asexual and sexual reproduction. More broadly, STRIPAK integrates signaling pathways that govern multiple aspects of development across fungal lineages, from vegetative growth to the formation of reproductive structures.

## How does STRIPAK regulate signaling pathways in fungi?

**Fig 2:**
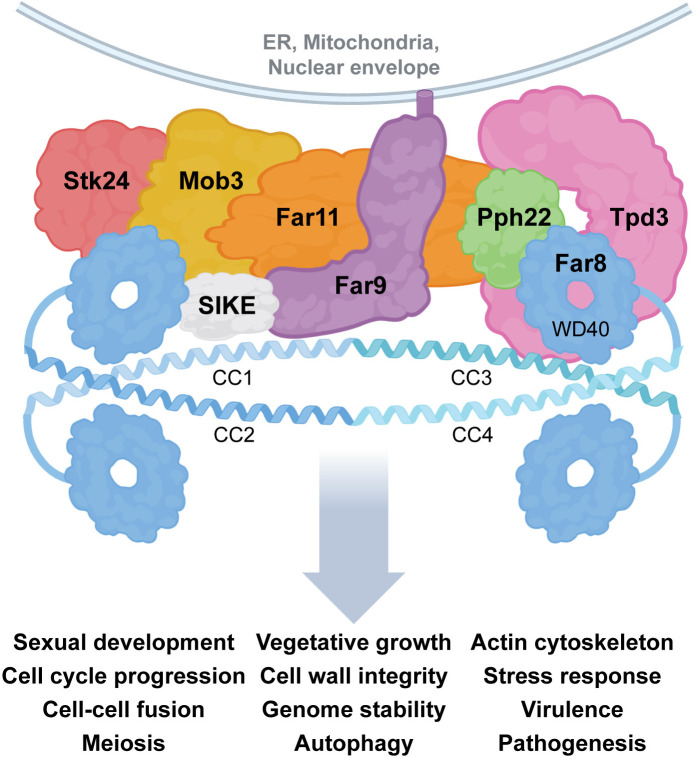
Structural model and functional roles of the fungal striatin-interacting phosphatase and kinase complex. A schematic model illustrates the organization and interactions of conserved fungal STRIPAK subunits, based on AlphaFold-Multimer predictions and published literature [9,18]. The striatin subunit Far8 forms a homotetramer via coiled-coil domains (CC1–CC4), serving as a scaffold for complex assembly. The WD40 domains of Far8 extend outward to mediate substrate interactions. The membrane-anchored protein Far9 localizes the complex to organelle membranes, including the endoplasmic reticulum, mitochondria, and nuclear envelope. Additional interacting components, including the PP2A catalytic subunit (Pph22), scaffold subunit (Tpd3), STRIP homolog (Far11), Mob3, SIKE, and a GCK family kinase (Stk24), complete the core STRIPAK complex. STRIPAK carries out conserved functions across fungi, including regulation of sexual and asexual development, cell growth and proliferation, and virulence, by acting on key signaling pathways and substrates. Schematic was partially created with BioRender.com [38].

The STRIPAK complex acts as a critical regulatory hub that integrates kinase and phosphatase signaling to control myriad aspects of fungal growth, development, and proliferation [21] (Fig 2). STRIPAK has been shown to influence the activity of the highly conserved mitogen-activated protein (MAP) kinase cascades that drive the pheromone response and cell wall integrity (CWI) pathways. In the unicellular yeast *Saccharomyces cerevisiae,* the STRIPAK homolog factor arrest (FAR) complex is required after pheromone exposure for cell cycle arrest in preparation for mating. In this example, the FAR complex controls actin cytoskeleton formation through the target of rapamycin complex 2 (TORC2) pathway [22–24]. The STRIPAK counterpart in *Schizosaccharomyces pombe*, the SIN (septin initiation network) inhibitory PP2A (SIP) complex, is important for cytokinesis as part of the sexual cycle [25]. In the filamentous fungus *N. crassa,* which has long served as a model to study cell-cell fusion, STRIPAK localization to the nuclear envelope is required for activation of the MAP kinase in the CWI pathway [17]. Direct protein-protein interactions between STRIPAK components and MAP kinases have also been identified in *S. macrospora,* highlighting STRIPAK’s roles in linking signaling pathways during development [26]. Quantitative phospho-proteomic analyses have further revealed a broad set of proteins involved in growth and metabolism whose phosphorylation depends upon a functional STRIPAK complex, underscoring its influence on fungal signaling dynamics [27,28]. These findings suggest that STRIPAK orchestrates signaling from specific subcellular sites, enabling precise control of phosphorylation-dependent pathways involved in fungal development and stress responses.

## How does STRIPAK interact with other cellular complexes or pathways in fungi?

STRIPAK interacts with multiple cellular signaling pathways involved in fungal development, growth, and stress adaptation. A key function of STRIPAK across fungi is to control PP2A phosphatase activity by directing it to specific subcellular compartments. In *S. cerevisiae*, the STRIPAK complex homolog modulates TORC2 signaling at the endoplasmic reticulum (ER) to regulate actin cytoskeleton organization; at the mitochondrial membrane, STRIPAK suppresses mitophagy, the selective autophagic degradation of mitochondria, through direct interactions with autophagy machinery [23,24,29]. In *S. pombe,* the STRIPAK homolog localizes to the spindle pole bodies during mitosis, where it spatially restricts SIN activity to ensure proper cytokinesis [30]. In *S. macrospora* and *N. crassa*, STRIPAK components are enriched at the nuclear envelope and are important for nuclear positioning and cell-cell communication during sexual development, likely by directing signaling pathways that coordinate nuclear dynamics [17,31]. Similarly, in *A. nidulans*, STRIPAK is localized to the nuclear envelope and promotes mitogen-activated protein kinase (MAPK) activation [14]. The conserved patterns of subcellular localization of STRIPAK to the ER, nuclear envelope, and mitochondria have also been observed in mammalian cells, suggesting that STRIPAK may coordinate similar cellular processes across eukaryotes [32]. These observations support the idea that STRIPAK functions as a scaffold that bridges organelles and integrates signaling between them, though the specific roles of its differential localization remain to be fully elucidated.

STRIPAK complex components also play critical roles in mediating responses to environmental and physiological stress. In *A. nidulans*, deletion mutations in STRIPAK components result in increased sensitivity to oxidative and cell wall stress [14]. In *Fusarium* species, disruption of STRIPAK alters colony morphology, pigmentation, and mycelial growth, particularly under nutrient-limiting conditions, suggesting a role in nutrient sensing and metabolic adaptation [33,34]. Similarly, in *C. neoformans,* STRIPAK mutants exhibit severe growth defects under nutrient stress and display increased sensitivity to elevated temperature, a key virulence-associated trait [18]. While in most fungi the loss of any STRIPAK component leads to similar phenotypes, the deletion of the *MOB3* subunit conferred increased stress tolerance and enhanced vegetative growth in both *C. neoformans* and *A. nidulans*, suggesting that individual subunits may have specialized roles or participate in subcomplexes with specialized functions.

## How does STRIPAK contribute to fungal virulence and host–pathogen interactions?

STRIPAK homologs have been directly implicated in fungal virulence and pathogenesis across diverse species. In the plant pathogens *Fusarium* and *Colletotrichum graminocola*, striatin orthologs are essential for host tissue penetration and colonization, which are important steps in establishing infection and promoting disease in the plant [13,35]. These effects are thought to involve formation of specialized infection structures and regulation of signaling pathways that coordinate hyphal growth and host recognition. STRIPAK also contributes to virulence-associated processes in the nematode trapping fungus *Duddingtonia flagrans* [36,37]. In these fungi, constricting rings, specialized structures dependent on hyphal fusion and intercellular communication, are formed in response to pheromone secreted by the nematodes. In *D. flagrans*, deletion of a STRIPAK component resulted in slow growth, abnormal hyphal morphology, and impaired trap formation. In the human pathogen *C. neoformans*, a functional STRIPAK complex has also been shown to be required for virulence*.* Strains carrying deletion mutations in PP2A B and C subunits exhibit defects in melaninization and polysaccharide capsule formation, two important virulence factors, and are attenuated for virulence in a murine inhalation model of infection [18]. In contrast, loss of the *MOB3* subunit led to hypervirulence and increased tolerance to host-associated stresses such as elevated temperature and CO_2,_ as well as increased melanin and capsule production, and higher fungal burden in infected tissues. STRIPAK similarly influences production of secondary metabolites that contribute to pathogenicity, such as mycotoxins, by modulating global regulators of these biosynthetic pathways [14,34]. Together, these findings suggest that while core STRIPAK components are generally required for pathogenicity, individual subunits can play unique and sometimes opposing roles in modulating virulence traits, potentially by influencing distinct downstream signaling effects within the broader STRIPAK network.

## What makes STRIPAK a promising target for antifungal strategies or a model for understanding fungal pathogenesis?

STRIPAK represents a compelling target for antifungal strategies and a valuable model for dissecting fungal pathogenesis due to its unique combination of conservation and functional specificity. While STRIPAK is broadly conserved across eukaryotes, its roles in fungi are highly context-dependent, often regulating developmental processes, stress adaptation, and virulence in species-specific ways [10]. For example, in *C. neoformans,* STRIPAK promotes thermal tolerance and virulence factor production critical for mammalian infection, while in the plant pathogen *F. graminearum*, STRIPAK primarily controls hyphal development and sexual reproduction, highlighting species-specific roles linked to their distinct pathogenic lifestyles [18,34]. Furthermore, fungal STRIPAK subunits exhibit sequence and domain variation relative to their mammalian counterparts [21], and phylogenetic comparisons illustrate both conservation and divergence across fungal lineages and humans (Fig 1). This divergence suggests that fungal STRIPAK complexes may have evolved specialized signaling mechanisms that could be selectively targeted without perturbing the host counterpart. Moreover, structural differences in key STRIPAK subunits and interactions may provide unique interfaces for targeting. For example, species-specific protein-protein interactions, posttranslational modifications, or subcellular localizations of STRIPAK subunits offer potential entry points for the development of antifungal agents that minimize off-target effects and toxicity to the mammalian host. At present, no STRIPAK-specific inhibitors in fungi have been reported, nor structural analyses of subunits identifying druggable sites. Future high-throughput screens and structural studies will be essential for assessing STRIPAK’s therapeutic potential.

## Conclusions and future directions

STRIPAK serves as a powerful model for understanding the integration of multiple signaling pathways that control fungal development. As a major conductor orchestrating developmental and stress-responsive networks, STRIPAK provides insight into how fungal pathogens coordinate complex cellular pathways. Ultimately, investigations on STRIPAK offer a broader framework for understanding the molecular basis of fungal growth, proliferation, and pathogenesis. Moving forward, elucidating the precise molecular mechanisms underlying fungal-specific STRIPAK functions and their interactions with other signaling modules will deepen our understanding of how this complex integrates environmental and developmental cues. Continued studies in fungal models will identify direct STRIPAK targets and regulatory dynamics, offering insights into conserved developmental processes across eukaryotes.
